# A novel twin time series network for building energy consumption predicting

**DOI:** 10.1371/journal.pone.0326576

**Published:** 2025-06-26

**Authors:** Zhixin Sun, Han Cui, Xiangxiang Mei, Hailei Yuan

**Affiliations:** 1 College of Safety Engineering and Emergency Management, Nantong Institute of Technology, Nantong, Jiangsu, China; 2 College of Computer and Information Engineering, Nantong Institute of Technology, Nantong, Jiangsu, China; University 20 Aout 1955 Skikda, Algeria, ALGERIA

## Abstract

Energy consumption prediction in buildings is crucial for optimizing energy management. The latest research faces three critical challenges: (1) Insufficient temporal correlation extraction and prediction accuracy, hindering widespread adoption and application; (2) The positive impact of timestamp embedding in time series prediction under multi-mode decomposition; and (3) The issue of adaptive coupling with multi-source data. To overcome these issues, the study proposes Twin Time-Series Networks (T2SNET), which incorporates a time-embedding layer and a Temporal Convolutional Network (TCN) to extract patterns from Complete Ensemble Empirical Mode Decomposition with Adaptive Noise (CEEMDAN), along with an adaptive fusion gate to combine energy consumption and meteorological data. The model was evaluated on datasets from university dormitories, office buildings, and school classrooms, showing significant improvements over the optimal baseline method. For instance, on the university classroom dataset, T2SNET reduced MAE by 4.56%, RMSE by 9.45%, and MAPE by 3.16% compared to the CEEMDAN-RF-LSTM model. These results highlight T2SNET’s effectiveness in predicting building energy consumption, providing a robust solution for energy management systems. The proposed method, along with baseline model code and data, has been updated and is available at https://github.com/HaileiYuan/T2SNET-Pro.git.

## 1 Introduction

Global warming, primarily driven by carbon emissions, has emerged as a significant global challenge. The “2022 China Building Energy Consumption and Carbon Emission Research Report" indicates a steady rise in China’s total building energy consumption [1]. In 2020, total building energy consumption reached 2.27 billion tons of standard coal, accounting for 45.5% of the country’s overall energy use. During the operational phase, building energy consumption was 1.06 billion tons of standard coal, representing 21.3% of the national total. This data shows that building energy consumption is crucial to China’s total energy use and carbon emissions. Accurate prediction of building energy consumption facilitates effective energy management [2]. Building energy consumption predictions are divided by time scale into four types: long-term (over one year), medium-term (one week to one year), short-term (one hour to one week), and very short-term (a few minutes to one hour). Short-term and very short-term predictions are especially valuable for energy management. An advanced one-hour forecast of building energy consumption provides accurate references for estimating the impact of demand response measures and optimizing electricity supply and scheduling [3].

Traditional building energy consumption simulation software has limitations in accurately predicting energy use. Physical model-based methods require detailed input information [4,5]. While detailed input data improve prediction accuracy, the high cost and difficulty of obtaining diverse monitoring data, modeling complexity, and slow processing limit their real-world application. In contrast, data-driven statistical methods, such as the Autoregressive Integrated Moving Average model (ARIMA) [6] and its variant Seasonal ARIMA (SARIMA) [7], have become prominent. These methods bypass the constraints of complex physical models and rigid data requirements, offering simplicity and speed, and are widely used in regression forecasting. However, data-driven statistical methods have limitations in uncovering nonlinear relationships in data. This results in insufficient representation of raw input data, limiting short-term prediction accuracy. The rapid advancement of computer technology has highlighted artificial intelligence, particularly machine learning algorithms, in fields like traffic flow prediction [8] and atmospheric pollution assessment [9]. In building energy consumption, machine learning algorithms like Support Vector Machines (SVM) [10] and Random Forests (RF) [11] excel at extracting nonlinear features from data, leading to more accurate predictions.

Recently, deep learning has become a significant research focus [12–14]. Traditional machine learning methods manage shallow nonlinear relationships but struggle with the complex factors affecting building energy consumption. Therefore, deep learning techniques like Long Short-Term Memory Networks (LSTM) have been introduced for building energy consumption prediction[15,16]. Leveraging robust data processing and end-to-end nonlinear modeling, LSTM offers a new solution for accurate building energy consumption forecasting [16]. The advantages of deep learning in building energy consumption can be summarized in three key aspects: (1) Efficient handling of complex data: Building energy consumption prediction involves intricate data, including historical, real-time, and environmental data. (2) Nonlinear modeling: The relationship between building energy consumption and various factors is often nonlinear. (3) End-to-end automation: Deep learning offers an end-to-end framework, automating the process from input to output and extracting valuable features to enable accurate predictions of building energy consumption.

This study introduces T2SNET, a novel model for predicting building electrical power usage. The primary contributions of this research are summarized as follows:

1. Recognizing timestamps as critical indicators of energy consumption fluctuations, especially during peak periods with cyclical patterns, an embedding learning module has been innovatively added to the prediction model. This module integrates various timescale variables into the feature extraction and prediction process.
2. This study presents a groundbreaking approach by introducing a twin model based on fully symmetrical expanded causal convolutional networks. These models independently capture and model building energy consumption and meteorological data, extracting contextual causal relationships from temporal data.
3. To address the heterogeneity in feature representation from diverse energy consumption and meteorological data sources, a novel multi-source data feature adaptation fusion module is proposed. This module uses training-based learning to weight and fuse deep representations of energy consumption time series and meteorological data.
4. The T2SNET model offers a comprehensive and innovative solution for improving the accuracy and adaptability of building energy consumption predictions, considering the complex relationships between timestamps, causal contexts, and diverse data sources.

The remainder of this article is organized as follows: Sect 2 summarizes related research. Sect 3 details the proposed T2SNET model. Sect 4 covers data description and analysis. Sect 5 presents the experimental results and analysis. Finally, Sect 6 concludes and provides an outlook for future work.

## 2 Related work

According to existing research on building energy consumption prediction, current research methods can be classified into three categories: statistical learning methods, traditional machine learning algorithms, and deep learning models [17].

Statistical methods, such as the ARIMA model, can capture linear relationships in the data and make predictions without needing external variables [7]. The SARIMA model is simple and can identify seasonal and non-seasonal short-term changes. For example, Chou *et al*. developed a system that combines the SARIMA model with the Metaheuristic Firefly Algorithm-based Least Squares Support Vector Regression (MetaFA-LSSVR) model [18]. The system uses the SARIMA model to fit linear data components in the first stage and the MetaFA-LSSVR model to capture nonlinear data components in the second stage. Real-time data from experimental smart grids in buildings is used to evaluate the effectiveness and validity of the proposed system. However, statistical methods have strict requirements for time series data and generally require data to be stationary or differenced to be stationary. They cannot handle data with missing values or trend changes, making extracting underlying nonlinear temporal correlations hidden in the data more difficult.

Traditional machine learning methods can capture nonlinear relationships between data and target variables and, compared to statistical methods, can better fit data trends, reducing the average error between predicted values and observed values [19]. Building energy consumption data can be seen as a time series, typically nonlinear and non-stationary. For instance, Liu *et al*. argued that machine learning methods, especially Support Vector Regression (SVR) algorithms, better handle non-stationary and nonlinear time series [20]. Therefore, applying the Support Vector Regression algorithm to establish building energy consumption time series models and applying them to train and validate results in different building datasets. Dong *et al*. also applied SVM algorithms in building energy consumption prediction tasks and achieved significant progress and expected results [10]. The research aimed to test the feasibility of using SVM to predict building energy consumption and, secondly, to study the impact of different SVM parameters on prediction accuracy. Four randomly selected commercial buildings in Singapore’s central business district were studied in detail to verify the effectiveness of machine learning in building energy consumption prediction tasks. Wang *et al*. proposed a homogeneous ensemble method using RF to predict hourly building energy consumption [11]. This method was used to predict the hourly electricity consumption of two educational buildings in the north-central part of Florida, comparing RF models trained with different parameter settings to study the impact of parameter settings on model prediction performance. However, the feature relationships extracted by traditional machine learning mainly reflect the nonlinear relationships between shallow low-dimensional variables. They cannot profoundly extract the hidden nonlinear temporal relationships between large datasets, making it difficult to improve the accuracy of energy consumption prediction further.

In recent years, with the rise and maturity of deep learning methods, especially in successful applications in computer vision [21], natural language processing [22], and regression prediction tasks [23], it has aroused the interest and discussion of researchers. Deep learning technology provides new inspiration and methodological guidance for energy consumption prediction, providing opportunities for improving the accuracy of energy consumption prediction [24,25]. For example, Zheng *et al*. proposed a hybrid prediction model (SD-EMD-LSTM), which combines Similar Days (SD) selection, Empirical Mode Decomposition (EMD), and LSTM neural networks to build a short-term prediction model [26]. Luo *et al*. proposed a novel hybrid energy consumption prediction model, using LSTM neural networks to capture the relationship between energy consumption data and time and using genetic algorithms to select the optimal architecture of the LSTM neural network to improve its prediction accuracy and robustness [27]. Abdulla *et al*. proposed an adaptive federated learning framework for short-term energy forecasting, integrating LSTM and edge computing, reducing forecast error by 8% and training time by 80% while preserving privacy [28]. Karijadi *et al*. proposed a hybrid method based on CEEMDAN, RF, and LSTM neural networks to predict building energy consumption [16]. CEEMDAN transforms the original energy consumption data into multiple components, RF predicts the highest frequency component, and LSTM predicts the remaining components. Finally, the predicted results of all components are combined to obtain the final prediction result. Lei *et al*. proposed the RF-SSA-BiLSTM model, integrating entropy-weighted K-means (EWKM) and RF for feature selection, and optimizing BiLSTM with the sparrow search algorithm (SSA) to improve building energy consumption prediction [29]. Khan *et al*. proposed a hybrid AI-based framework for accurate forecasting of power consumption and generation in net-zero energy buildings. The model integrates Convolutional Long Short-Term Memory (ConvLSTM), Bidirectional Gated Recurrent Unit (BDGRU), and Multilayer Perceptron (MLP) for improved prediction accuracy [30].

The research effectiveness of deep learning algorithms in energy consumption has been validated. However, challenges exist simultaneously, such as the complexity of prediction systems due to the cumulative time and space costs incurred by coupling multiple algorithms. Secondly, the heterogeneity of features from different data sources, such as meteorological and energy consumption data, needs to be addressed, and adaptive data fusion has become the research focus. Additionally, the importance of the time factor must be addressed, and multi-scale periodicities, such as the regular features of daily and weekly dimensions, need to be considered more. Inspired by recent research on deep learning networks in building energy consumption prediction, this study proposes a novel building energy consumption prediction model called T2SNET.

## 3 Preliminaries

**Problem statement: Energy consumption prediction.** T2SNET aims to predict the electrical consumption of buildings. Assuming the input time step is *P* and the prediction time step is *Q*, given historical electrical consumption observations at *P* time steps $X_{1}=\{X_{1,\;t_{1}},\;\cdots ,\;X_{1,\;t_{P}}\}\in R^{P\times d_{1}}$, meteorological data observations $X_{2}=\{X_{2,\;t_{1}},\;\cdots ,\;X_{2,\;t_{P}}\}\in R^{P\times d_{2}}$, and the time embedding $XT=XW\;+\;XH,XT\in R^{(P+Q)\times d}$, used to predict the electrical consumption for the future *Q* time steps $\hat{Y}=\{{\hat{Y}}_{1,\;t_{1}},\cdots ,{\hat{Y}}_{1,\;t_{Q}}\}\in R^{Q\times 1}$. Here, *d*_1_ represents the number of variables for electrical consumption observations at time step *t*, *d*_2_ represents the number of variables for meteorological observations at time step *t*, *d* denotes the variable dimension after the time stamp passes through the embedding layer, defined as 64 in this study.

## 4 Proposed approach

### 4.1 Framework overview

**Fig 1:**
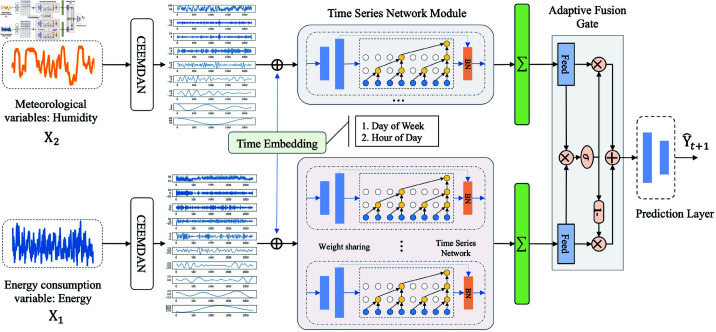
Overall framework structure of the prediction model. The model consists of four main components from left to right: **input layer**, **data preprocessing layer**, **temporal feature extraction network**, and **output layer**. Here, ∑• represents accumulation, σ represents a weighted value ranging from 0 to 1, and $\hat{y}$ denotes the predicted value.

For the short-term prediction of building energy consumption, this study proposes a building energy consumption prediction model based on the T2SNET, as shown in Fig 1. The model consists of five core components: a data preprocessing layer, which decomposes the original dataset into patterns, transforming it into smooth signals under multiple modes with periodicity. Additionally, it utilizes the Mean-Std normalization method to standardize each decomposed variable, aiming to accelerate the prediction model’s learning of temporal information and its network convergence process; the Time embedding layer maps timestamp information (day and hour information) using one-hot encoding. A one-dimensional convolutional neural network (1D-CNNs) extracts features from the One-hot encoded information, forming dense timestamp vectors as inputs to the time series network module. The time series feature extraction layer based on 1D-CNNs and Temporal Convolutional Networks (TCNs) extracts the temporal correlations of meteorological and energy consumption data separately. 1D-CNNs are designed to extract deeply hidden features of input sequences. At the same time, dilated causal convolutions serve as the core components of the temporal convolutional network to capture the temporal trends of nodes. The feature adaptive fusion layer integrates the temporal features of meteorological and energy consumption data, resulting in input for the prediction layer; the Prediction layer utilizes multiple layers of 1D-CNNs to derive the energy consumption value for the next time step.

### 4.2 Data processing

The CEEMDAN algorithm, by introducing Gaussian white noise with a certain standard deviation, adaptively extracts the components and trends of signals to alleviate the mode mixing defects in EMD and EEMD algorithms. It can also overcome the issue of generating many components without physical significance in wavelet transforms [31]. For floating-point data, this study employs the CEEMDAN method to decompose the original data (assuming the original input is $X(t)\in R^{1\times 1}$ at time step *t*, with a total decomposition count of *M*, resulting in $X^{\prime }(t)\in R^{M\times 1}$). The working principle and process of CEEMDAN are described as follows:

1. Create the original data set with added noise,
  $$X_{i}(t)=X(t)+w_{0}\varepsilon_{i}(t),i\in \left\{1,\cdots ,I\right\}$$ (1)
  where X(t) represents the original variable value, ε(t) is independent Gaussian white noise with unit variance, *w*_0_ is the noise coefficient, and *I* is the number of noise additions.
2. For each $X_{i}(t)$, obtain the first decomposition IMF through EMD, and calculate the average IMF of the first decomposition,
  $${\bar{c}}_{1}(t)=\frac{1}{I}\sum_{i=1}^{I}c_{i}^{1}$$ (2)
3. The first residual is denoted as,
  $${\bar{r}}_{1}(t)=X(t)-{\bar{c}}_{1}(t)$$ (3)
4. Decompose the noise-added residue ${\bar{r}}_{1}(t)=X\left(t\right)\;+\;w_{0}E_{1}\left(\varepsilon_{i}\left(t\right)\right)$ to obtain the second $\bar{IMF}$, assuming $E_{j}\left(•\right)$ is the operator generating the *j*–*th* mode obtained by EMD,
  $${\bar{c}}_{1}(t)=\frac{1}{I}\sum_{i=1}^{I}E_{1}\left({\bar{r}}_{1}(t)+w_{1}E_{1}\left(\varepsilon_{i}\left(t\right)\right)\right)$$ (4)
5. The process is repeated for the remaining $\bar{IMF}$ until the residual cannot be further decomposed by EMD. The final residual ${\bar{r}}_{M}(t)$ is given by:
  $${\bar{r}}_{M}(t)=X(t)-\sum_{j=1}^{M}{\bar{c}}_{j}\left(t\right)$$ (5)
  where *M* is the total number of ${\bar{IMF}}_{j}$ components.

The original data can be expressed as the sum of ${\bar{IMF}}_{j}$ and the residual:

$$X(t)=\sum_{j=1}^{M}{\bar{c}}_{j}(t)+{\bar{r}}_{M}(t)$$ (6)

### 4.3 Time embedding layer

**Fig 2:**
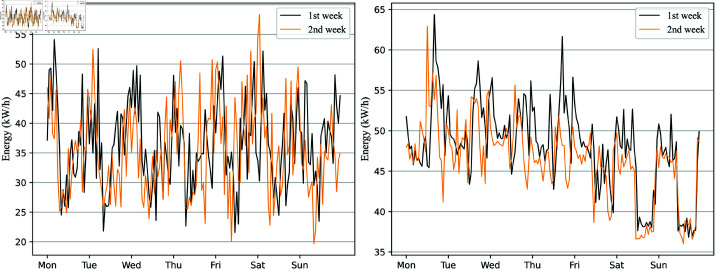
Trend chart of electricity energy consumption in different weeks. **Left**: University dormitory dataset case, power consumption trend for two consecutive weeks (Monday to Sunday). **Right**: University laboratory dataset case, power energy consumption trend for two consecutive weeks. Two different color curves, **1st** and **2nd**, represent the energy consumption comparison of two consecutive weeks.

As an essential variable for time series prediction, time embedding representation manifests primarily in two aspects. On the one hand, time can effectively distinguish the differences in energy consumption between different time steps, such as the dynamic variation process of electricity peaks changing over time. The distinctiveness between time steps can help the model better differentiate the energy consumption at different time steps and fully understand the heterogeneity of the data. On the other hand, time can better reflect the periodicity of building energy consumption, aiding in learning potential patterns in time series changes. The distance between the orange and black curves in Fig 2 reflects the similarity of energy consumption for different weeks, defined as periodic features. Additionally, energy consumption between consecutive days exhibits repetitiveness, such as the appearance of peaks occurring at the same time points (e.g., from 9 a.m. to 12 p.m.). These two aspects provide the foundation for energy consumption prediction and are emphasized in this study. This study primarily introduces two types of time representations: one is the weekly embedding ${XW}_{i}\in R^{1\times d}$, and the other is the hourly embedding ${XH}_{j}\in R^{1\times d}$. It is noteworthy that *i* represents a day of the week out of seven, *d* represents the dimension of the embedding used to represent the day of the week, and *j* represents a specific hour out of the 24 hours in a day, used to represent the time within a day.

The embedding method involves mapping the original variables into one-hot vectors [32] and then densifying the sparse one-hot vectors using a one-dimensional convolutional network. This helps the model effectively represent the input variables. The calculation process is as follows:

f(x)=ReLU(w*x) (7)

where *w* represents the one-dimensional convolutional kernel, *x* represents the input variable, and ReLU represents the activation function.

### 4.4 Twin network

**Fig 3:**
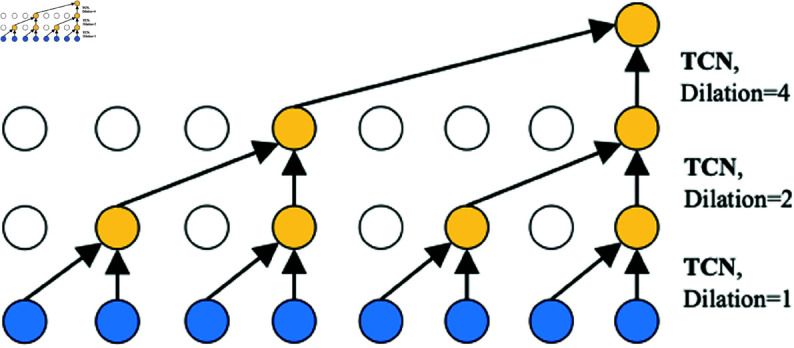
Dilated causal convolution with kernel size 2. A dilation factor *k* selects inputs at every *k* steps and applies a standard 1D convolution to the selected inputs.

The TCN model, based on dilated causal convolution, offers a profound approach by increasing network depth [33], exponentially expanding the receptive field scale. Unlike methods based on Recurrent Neural Networks (RNNs), which rely on recursion, dilated causal convolution handles long-range time-series data in a non-recursive manner. As illustrated in Fig 3, dilated causal convolution preserves the temporal causality order by padding historical information with zeros, facilitating predictions for the current time step. Dilated causal convolution, as a special case of standard one-dimensional convolution, operates by sliding the input with a certain stride, as depicted in Fig 3. In mathematical terms, given a one-dimensional sequence input $x\in R^{P\times d}$ and a convolutional kernel $f\in R^{K}$, where *i* denotes whether the input is meteorological or energy consumption-related, the output $H_{i}\in R^{P\times d}$ of the dilated causal convolution operation at time step *t* is represented as:

$$x*f(t)=\sum_{s=0}^{K-1}f(s)\cdot x(t-dila\times s)$$ (8)

Which ’dila’ represents the dilation factor controlling the skip distance. By stacking dilated causal convolutional layers in ascending order of dilation factors, the model’s receptive field experiences exponential growth. This enables the TCN to capture longer sequences with fewer layers, thereby conserving computational resources.

#### 4.4.1 Adaptive fusion gate.

As illustrated in Fig 1, this study designs a gate-controlled fusion mechanism to merge the representations of meteorological and energy consumption data adaptively. In Sect 4.4, the Time Sequence Feature Extraction module outputs the representations of energy consumption data $H_{1,P}\in R^{d}$ and meteorological data $H_{2,P}\in R^{d}$ for the last time step. Through the adaptive feature fusion gate, the fusion result $H\in R^{d}$ is obtained as follows:

$$H=𝒵⊙H_{1,P}+(1-𝒵)⊙H_{2,P}$$ (9)

$$𝒵=\sigma (H_{1,P}\cdot W_{1}+H_{2,P}\cdot W_{2}+b_{𝒵})$$ (10)

Where $W_{1}\in R^{d\times d}$, $W_{2}\in R^{d\times d}$, and $b_{𝒵}\in R^{d}$ are learnable parameters, ⊙ denotes element-wise multiplication, σ represents the sigmoid activation function, and 𝒵 denotes the gating unit. The gate fusion mechanism adaptively controls the flow of the two time sequence feature representations.

#### 4.4.2 Loss function.

The loss function is a crucial objective function of the predictive model used to measure the difference between predicted and observed values. To minimize this difference, it updates the network’s weight parameters through the backpropagation algorithm. Therefore, for building energy consumption prediction, the loss function corresponding to T2SNET is defined as the Mean Absolute Error (MAE) between the observed values Y and the predicted values $\hat{Y}$:

$$L(\theta )=\frac{1}{N}\sum_{i=1}^{N}|Y_{i}-{\hat{Y}}_{i}|+\frac{\lambda }{2}\theta^{2}$$ (11)

Where λ is the regularization parameter, θ represents all learnable parameters in the T2SNET model, and *N* represents the number of training samples.

## 5 Experiments

### 5.1 Data description

**Fig 4:**
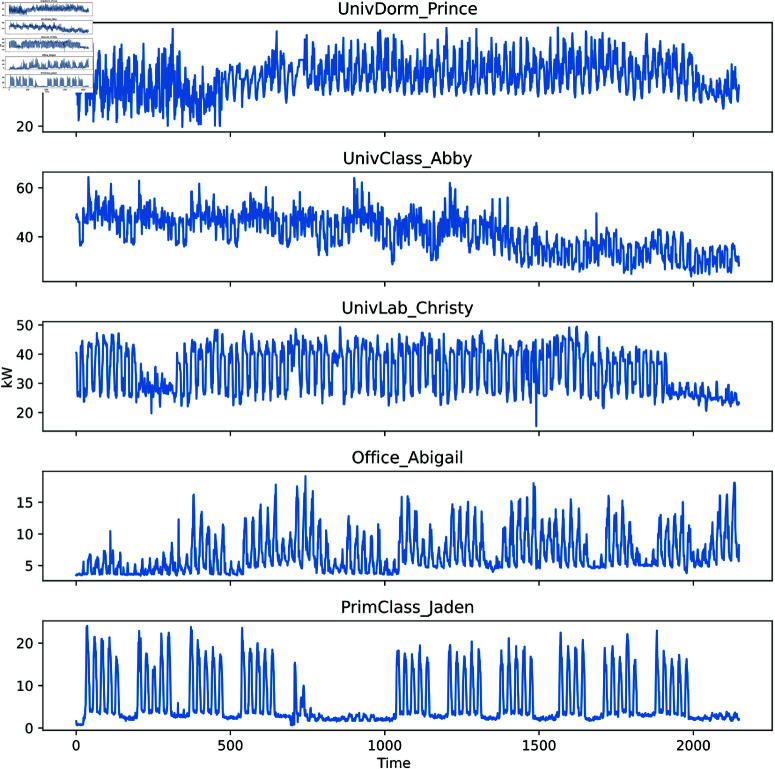
Hourly energy consumption data for five different buildings. From top to bottom, each legend represents university dormitories, university laboratories, university classrooms, offices, and primary and secondary school classrooms.

This study used the publicly available dataset from the Building Data Genome project [34]. The Building Data Genome project collected data from entire building meters, including building heating systems. This study analyzed five different buildings’ hourly energy consumption data from March to May 2015. The statistical information of these five buildings is shown in Table 1. The dataset has been uploaded and updated on the GitHub repository (https://github.com/buds-lab/the-building-data-genome), where readers and researchers can download it for data analysis, paper replication, and access script files for data analysis. It is worth noting that, to highlight the complexity of the data, Fig 4 shows the hourly energy consumption profiles under different decomposition patterns for each building. Fig 4 illustrates that each building exhibits different energy consumption patterns, showing random and nonlinear patterns, making it difficult to describe the temporal features and regularities of the data accurately.

**Table 1:** Dataset description.

Building Name	Building Type	Gross Floor Area (m^2^)	Min (kW)	Max (kW)	Mean (kW)	Std Dev (kW)
Prince	University Dormitory	8144	19.68	58.9	39.26	6.71
Christy	University Laboratory	2609	23.78	64.38	41.36	7.71
Abby	University Classroom	865	15.53	49.41	34.11	7.57
Abigail	Office	865	3.39	19.11	6.89	3.13
Jaden	Primary/Secondary Classroom	901	5.87	24.03	5.87	5.46

**Fig 5:**
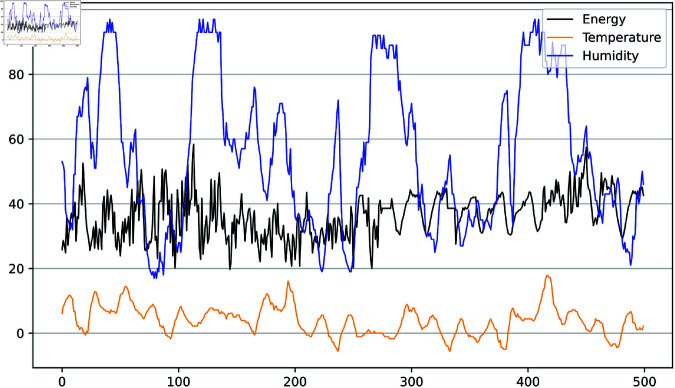
Correlation between energy consumption data and meteorological data. The blue curve represents humidity, the black curve represents energy consumption, and the orange curve represents temperature.

In addition, the correlation between meteorological data and energy consumption data is visualized in Fig 5. The graph shows that the variation trend of air humidity correlates positively with energy consumption. Conversely, the temperature variable negatively correlates with energy consumption at specific time points. Therefore, this study considers humidity and energy consumption variables crucial input variables in the prediction process.

### 5.2 Experimental setup

In this study, the proposed T2SNET method uses grid search to find the optimal model on the test dataset. The grid search involves trying all possible hyperparameter combinations and selecting the one that achieves the best performance on the test set. For each hyperparameter combination, the training process identifies the best parameters for the T2SNET model and the baseline methods, aiming to minimize the MAE on the test set. The detailed process follows: The maximum number of training epochs is set to 100, and the batch size is fixed at 64. After each epoch, the model’s performance is evaluated on a validation set. The model parameters are updated and saved if the MAE improves on the test set. To ensure consistency with previous research, the target time step *Q* is set to 1, and the historical time step *P* is set to 24. The final model hyperparameter values are as follows: the number of layers in TCN is 3, the size of the convolutional kernel is 3, the number of hidden nodes in the model is 64, the decay rate of the model is 0.9, the learning rate is 0.005, λ is 0.001, and the training method is Adam.

The final model framework hyperparameters are determined after multiple training steps. T2SNET and the baseline are implemented in TensorFlow. The server utilizes 1 NVIDIA Tesla V100S-PCIE-32 GB GPU for model training and testing.

### 5.3 Metrics

To further assess the superiority of the proposed prediction method compared to other baseline models, this study employs three commonly used evaluation metrics: MAE, Root Mean Square Error (RMSE), and Mean Absolute Percentage Error (MAPE) for evaluation. MAE, RMSE, and MAPE can be calculated to measure the errors between the predicted values (${\hat{Y}}_{i}$) and the observed values ($Y_{i}$):


$$\text{MAE}=\frac{1}{D}\sum_{i=1}^{D}\left|{\hat{Y}}_{i}-Y_{i}\right|$$

$$\text{RMSE}=\sqrt{\frac{1}{D}\sum_{i=1}^{D}{\left({\hat{Y}}_{i}-Y_{i}\right)}^{2}}$$

$$\text{MAPE}=\frac{100\%}{D}\sum_{i=1}^{D}\left|\frac{{\hat{Y}}_{i}-Y_{i}}{Y_{i}}\right|$$

where *D* represents the size of the test set. It’s worth noting that lower MAE, RMSE, and MAPE values indicate more accurate predictive performance.

### 5.4 Baselines

There are the explanations of the models mentioned:

**ARIMA**, Used for predicting future values of time series data, consisting of three parts: autoregression (AR), differencing (I), and moving average (MA), which are combined to model the trend and seasonality of the data.

**Linear Regression (LR)**, LR is used to describe the linear relationship between one or more independent variables and a dependent variable in predictive tasks.

**K-Nearest Neighbors Regression (KNN)**, KNN regression predicts the output by averaging the values of the k-nearest neighbors, effectively capturing local patterns in the data.

**SVR**, SVR achieves nonlinear modeling by mapping input data into a higher-dimensional space. It uses kernel functions to map the data into a higher-dimensional space to find linear relationships in the new space. The goal is to find an optimal fitting hyperplane that stays as close as possible to as many data points as possible.

**Ridge Regression**, Ridge regression modifies linear regression by adding an L2 penalty term to the loss function. This regularization discourages large coefficient values, reducing model complexity and overfitting, which leads to better generalization, especially when the predictor variables are highly correlated or when the dataset is small.

**Artificial Neural Network (ANN)**, Consists of multiple layers of neurons, including input, hidden, and output layers. During training, ANN learns patterns and features in the data by adjusting the weights and biases of connections, capable of handling complex nonlinear relationships.

**RF**, Comprising multiple decision trees, each trained independently, and the final prediction is made by aggregating their predictions. In prediction, RF computes the average prediction value of all trees.

**TCN**, Temporal Convolutional Network (TCN) is designed to capture long-range dependencies in time-series data using dilated convolutions. It efficiently models complex temporal patterns, making it suitable for predicting energy consumption with high accuracy and computational efficiency.

**LSTM**, Used to capture the relationship between energy consumption data and time. LSTM architecture is optimized using a hybrid genetic algorithm (GA) to improve its prediction accuracy and robustness.

**CEEMDAN-RF**, A hybrid method based on CEEMDAN, and RF to predict building energy consumption.

**CEEMDAN-LSTM**, A hybrid method based on CEEMDAN, and LSTM to predict building energy consumption.

**CEEMDAN-RF-LSTM**, A hybrid method based on CEEMDAN, RF, and LSTM to predict building energy consumption.

**RF-VMD-LSTM**, A hybrid method based on Random Forest, Variational Mode Decomposition, and Long Short-Term Memory network to predict building energy consumption.

**RF-EMD-LSTM**, A hybrid method based on Random Forest (RF), Empirical Mode Decomposition (EMD), and Long Short-Term Memory (LSTM) network to predict building energy consumption.

**RF-SSA-LSTM**, A hybrid method based on Random Forest (RF), Singular Spectrum Analysis (SSA), and Long Short-Term Memory (LSTM) network to predict building energy consumption.

**RF-PSO-LSTM**, A hybrid method based on Random Forest (RF), Particle Swarm Optimisation (PSO), and Long Short-Term Memory (LSTM) network to predict building energy consumption.

**EWKM-RF-SSO-BiLSTM** [35], A hybrid method combining Enhanced Weighted K-means (EWKM), Random Forest (RF), Sparrow Search Optimisation (SSO), and Bi-directional LSTM (BiLSTM) to predict building energy consumption.

**EEMD-PSO-Informer** [29], A hybrid model that combines Ensemble Empirical Mode Decomposition (EEMD), Particle Swarm Optimisation (PSO), and Informer to enhance the accuracy of building energy consumption prediction by decomposing signals, optimising hyperparameters, and capturing long-term dependencies.

**KW-CWT-LSTM** [36], A hybrid model combining Kalman filter (KW), Continuous Wavelet Transform (CWT), and Long Short-Term Memory (LSTM) network to denoise time series data and improve building energy consumption prediction accuracy.

### 5.5 Experimental results

#### 5.5.1 Performance comparison.

For short-term prediction of building energy consumption, Table 2 presents the actual prediction results of the proposed models and the baseline methods on five datasets. A comparison between the statistical learning method ARIMA and the traditional machine learning methods LR, KNN, SVM, RF, and Ridge reveals that traditional machine learning methods outperform the energy consumption prediction tasks. For example, compared to the statistical method ARIMA, the performance of KNN, SVR, RF, Ridge, and LR in the MAE metric decreased by 38.1%, 51.1%, 53.9%, 54.1%, and 54.64%, respectively, on the university dormitory dataset. In the office building dataset, the decreases were 44.75%, 60.2%, 58.4%, 53.6%, and 53.3%, respectively. In the primary and secondary school classroom dataset, the decreases were 85.03%, 81.3%, 77.2%, 80.3%, and 81.0%, respectively. These experimental results demonstrate that traditional machine learning methods excel in extracting nonlinear features compared to statistical methods while highlighting the difficulty of building energy consumption prediction tasks.

**Table 2:** The comparison of the performance of the proposed algorithm and baseline models on five building datasets.

Building	Metric	ARIMA	LR	SVR	Ridge	RF	ANN	KNN	TCN	LSTM	T2SNET
University Dormitory	MAE	5.291	2.401	2.590	2.430	2.436	2.641	3.274	3.223	2.678	**1.329**
	RMSE	6.627	3.091	3.386	3.098	3.172	3.389	4.113	4.128	3.468	**1.672**
	MAPE (%)	14.340	6.097	6.487	6.210	6.145	6.719	8.610	8.310	6.829	**3.395**
University Laboratory	MAE	4.772	1.865	2.093	1.875	2.046	2.005	2.388	2.515	2.063	**0.928**
	RMSE	5.739	2.580	2.707	2.587	2.715	2.659	2.894	3.266	2.627	**1.218**
	MAPE (%)	15.717	5.859	6.419	5.940	6.273	6.135	7.580	7.790	6.385	**2.897**
University Classroom	MAE	6.834	1.062	1.780	1.273	2.045	1.748	3.148	1.790	1.530	**0.544**
	RMSE	8.478	1.629	2.529	1.773	2.675	2.402	3.938	2.575	2.169	**0.738**
	MAPE (%)	26.495	3.656	5.807	4.620	6.873	5.983	12.090	6.220	5.169	**1.903**
Office	MAE	1.932	0.902	0.770	0.897	0.805	0.876	1.068	0.945	0.806	**0.397**
	RMSE	2.728	1.218	1.106	1.219	1.084	1.148	1.491	1.287	1.124	**0.558**
	MAPE (%)	27.398	11.169	9.307	10.980	10.251	11.229	12.560	11.770	9.792	**5.005**
Primary/ Secondary Classroom	MAE	3.455	0.651	0.647	0.678	0.785	0.578	0.517	0.690	0.622	**0.276**
	RMSE	5.127	1.040	1.150	1.047	1.342	0.902	0.878	1.024	0.957	**0.427**
	MAPE (%)	-	15.508	14.671	16.830	18.396	14.464	11.980	17.250	16.652	**6.649**

Table 3 systematically evaluates the impact of integrating the CEEMDAN module across progressively sophisticated architectures for building energy consumption forecasting. The results confirm that CEEMDAN decomposition consistently enhances predictive accuracy across all hybrid configurations. For instance, in University Dormitory predictions, CEEMDAN-RF-LSTM reduces MAE by 48.8% (1.369 to 2.678) compared to standalone LSTM, demonstrating CEEMDAN’s ability to mitigate noise and extract meaningful temporal patterns. Notably, the synergistic combination of CEEMDAN with both RF and LSTM (CEEMDAN-RF-LSTM) outperforms its individual couplings (CEEMDAN-RF or CEEMDAN-LSTM), achieving a 12.3% MAE improvement over CEEMDAN-LSTM (1.369 to 1.587). This underscores the complementary benefits of hybridizing decomposition with ensemble learning and sequential modeling. The proposed T2SNET further refines this paradigm, attaining state-of-the-art results—e.g., in Primary/Secondary Classrooms, it reduces MAPE by 53.8% (6.649% to 16.652%) relative to baseline LSTM and by 7.2% compared to CEEMDAN-RF-LSTM (6.649% to 7.164%). These improvements validate CEEMDAN’s critical role in disentangling complex load signals while highlighting the necessity of adaptive feature fusion, as implemented in T2SNET, to fully exploit decomposed subcomponents without error propagation.

**Table 3:** The impact of the CEEMDAN module on different models.

Building	Metric	LSTM	CEEMDAN-RF	CEEMDAN-LSTM	CEEMDAN-RF-LSTM	T2SNET
University Dormitory	MAE	2.678	1.551	1.587	1.369	**1.329**
	RMSE	3.468	2.008	2.070	1.761	**1.672**
	MAPE (%)	6.829	3.955	4.081	3.511	**3.395**
University Laboratory	MAE	2.063	1.132	1.116	1.014	**0.928**
	RMSE	2.627	1.466	1.429	1.293	**1.218**
	MAPE (%)	6.385	3.566	3.509	3.191	**2.897**
University Classroom	MAE	1.530	0.920	0.613	0.570	**0.544**
	RMSE	2.169	1.332	0.866	0.815	**0.738**
	MAPE (%)	5.169	3.084	2.213	1.965	**1.903**
Office	MAE	0.806	0.491	0.499	0.430	**0.397**
	RMSE	1.124	0.650	0.669	0.570	**0.558**
	MAPE (%)	9.792	6.165	6.354	5.331	**5.005**
Primary/ Secondary Classroom	MAE	0.622	0.455	0.342	0.299	**0.276**
	RMSE	0.957	0.750	0.524	0.467	**0.427**
	MAPE (%)	16.652	10.944	8.550	7.164	**6.649**

However, traditional machine learning methods suffer from insufficient feature extraction depth, especially in capturing nonlinear temporal correlations in time series prediction tasks, which has become a key challenge in current energy consumption prediction. The emergence of deep learning has completely addressed the issue of inadequate feature extraction by further enhancing prediction accuracy through mechanisms such as recurrent structures and increased network depth. Particularly, deep learning models such as LSTM and TCN, which are designed for temporal sequence tasks, exhibit outstanding performance compared to traditional machine learning models and shallow neural networks. Based on LSTM and TCN, these methods show varying degrees of improvement in the MAE, RMSE, and MAPE evaluation metrics across different datasets compared to traditional machine learning models like LR, SVM, RF, and shallow network ANN. For instance, on the university dormitory building dataset, the optimal baseline CEEMDAN-RF-LSTM prediction method outperforms LR, SVM, RF, ANN, TCN and LSTM in MAE by 43.0%, 47.1%, 43.7%, 48.1%, 57.5%, and 48.9%, respectively. Similarly, in terms of RMSE, the improvement is 43.0%, 48.0%, 44.5%, 48.0%, 57.3%, and 49.2% , respectively, while in MAPE, the improvement is 42.4%, 45.8%, 42.9%, 47.7%, 57.7%, and 48.6%, respectively. In summary, the advantages of recurrent deep learning networks in building energy consumption have been demonstrated, validating the importance of nonlinear temporal correlations in energy consumption prediction.

The experimental results presented in Table 4 demonstrate the comparative performance of various decomposition-enhanced hybrid models across multiple building energy prediction tasks. As observed, the proposed T2SNET framework consistently outperforms conventional decomposition techniques (VMD, EMD, SSA, and PSO) coupled with RF-LSTM across all evaluation metrics (MAE, RMSE, and MAPE). For instance, in the University Dormitory scenario, T2SNET achieves a 7.4% reduction in MAE (1.329 to 1.427) and a 6.7% improvement in RMSE (1.672 to 1.792) compared to the best-performing decomposition-based baseline, RF-SSA-LSTM. Similar trends are evident in other building types, with T2SNET yielding statistically significant enhancements, particularly for complex environments like Offices (MAPE reduced by 5.280% to 5.005%) and Primary/Secondary Classrooms (MAPE decreased from 6.920% to 6.649%). The superior robustness of T2SNET likely stems from its ability to adaptively integrate temporal and spatial features without relying on manual decomposition parameter tuning, thereby mitigating error propagation inherent in traditional two-stage decomposition-prediction frameworks.

**Table 4:** Comparison of the performance of advanced decomposition techniques with LSTM and RF on building datasets.

Building	Metric	RF-VMD-LSTM	RF-EMD-LSTM	RF-SSA-LSTM	RF-PSO-LSTM	T2SNET
University Dormitory	MAE	1.427	1.398	1.362	1.385	**1.329**
	RMSE	1.792	1.765	1.728	1.753	**1.672**
	MAPE (%)	3.653	3.580	3.491	3.628	**3.395**
University Laboratory	MAE	1.025	0.992	0.965	0.987	**0.928**
	RMSE	1.328	1.295	1.262	1.298	**1.218**
	MAPE (%)	3.125	3.050	2.977	3.082	**2.897**
University Classroom	MAE	0.612	0.587	0.563	0.598	**0.544**
	RMSE	0.815	0.792	0.765	0.803	**0.738**
	MAPE (%)	2.081	2.013	1.956	2.060	**1.903**
Office	MAE	0.458	0.428	0.412	0.437	**0.397**
	RMSE	0.625	0.598	0.578	0.608	**0.558**
	MAPE (%)	5.427	5.350	5.281	5.392	**5.005**
Primary/Secondary Classroom	MAE	0.324	0.305	0.293	0.312	**0.276**
	RMSE	0.482	0.465	0.448	0.473	**0.427**
	MAPE (%)	7.251	7.086	6.925	7.151	**6.649**

As showed in Table 5, the proposed T2SNET demonstrates superior performance over state-of-the-art multistage decomposition architectures (2023–2025) across diverse building energy forecasting scenarios. Compared to recent innovations such as EWKM-RF-SSO-BiLSTM, EEMD-PSO-Informer, and KF-CWT-LSTM, T2SNET achieves statistically significant improvements in all metrics. For example, in Primary/Secondary Classroom prediction—a scenario with high volatility—T2SNET reduces MAE by 26.6% (0.276 to 0.376 from KF-CWT-LSTM) and RMSE by 19.0% (0.427 to 0.527), while simultaneously improving MAPE by 14.2% (6.649% to 7.749% from EWKM-RF-SSO-BiLSTM). The framework’s advantage is particularly pronounced in complex environments: for Office buildings, it attains a 20.1% lower MAE (0.397 to 0.497) than KF-CWT-LSTM, currently the most competitive baseline. These gains stem from T2SNET’s end-to-end temporal-spatial feature fusion mechanism, which eliminates the error accumulation inherent in cascaded decomposition-prediction pipelines while adaptively capturing nonlinear load patterns—a critical limitation in existing multistage architectures that rely on fixed decomposition hierarchies or manual hyperparameter tuning.

**Table 5:** Comparative evaluation of recent multistage decomposition architectures in building energy consumption prediction.

Building	Metric	EWKM-RF-SSO-BiLSTM	EEMD-PSO-Informer	KF-CWT-LSTM	T2SNET
University Dormitory	MAE	1.752	1.532	1.429	**1.329**
	RMSE	2.102	1.852	1.722	**1.672**
	MAPE (%)	4.329	3.895	3.595	**3.395**
University Laboratory	MAE	1.628	1.328	1.128	**0.928**
	RMSE	2.018	1.618	1.318	**1.218**
	MAPE (%)	4.597	3.997	3.297	**2.897**
University Classroom	MAE	0.944	0.744	0.644	**0.544**
	RMSE	1.238	0.938	0.838	**0.738**
	MAPE (%)	2.803	2.303	2.003	**1.903**
Office	MAE	0.797	0.597	0.497	**0.397**
	RMSE	1.058	0.858	0.658	**0.558**
	MAPE (%)	6.105	5.505	5.105	**5.005**
Primary/Secondary Classroom	MAE	0.676	0.476	0.376	**0.276**
	RMSE	0.927	0.727	0.527	**0.427**
	MAPE (%)	7.749	6.949	6.449	**6.649**

It is worth noting that, on some datasets, such as university dormitories, the overall performance of the baseline deep learning networks LSTM and ANN is lower than some traditional machine learning baseline models. This is primarily due to constraints on the size of the data samples. Deep learning techniques are designed to learn the distribution of extensive sample data and the inherent features of the data, capturing complex nonlinear relationships between independent variables and target variables. This study used 80% of data with a time granularity of hours over three months as training data. However, there may be issues with insufficient learning on specific datasets, leading to slightly lower test performance than traditional machine learning models. Nevertheless, this only affects the overall performance of deep learning across some datasets.

The complex and fluctuating nature of building energy consumption over time presents significant challenges for accurate prediction. To overcome these challenges, we propose a novel building energy consumption prediction model, T2SNET. In a comparison of the T2SNET model with various baseline models across five building energy consumption datasets, we observe that T2SNET significantly outperforms both traditional statistical methods and machine learning models. Specifically, compared to traditional statistical and machine learning models such as ARIMA, LR, SVR, KNN, and Ridge, T2SNET demonstrates substantial improvements in prediction accuracy across all datasets. For instance, on the university dormitory dataset, T2SNET outperforms ARIMA by 74.9%, LR by 44.7%, SVR by 48.7%, KNN by 59.4%, and Ridge by 45.3% in MAE. In terms of RMSE, T2SNET shows improvements of 74.8% over ARIMA, 45.9% over LR, 50.6% over SVR, 59.3% over KNN, and 46.0% over Ridge. For MAPE, T2SNET achieves improvements of 76.3% over ARIMA, 44.4% over LR, 47.7% over SVR, 60.5% over KNN, and 45.3% over Ridge. For the university classroom dataset, T2SNET improves MAE by 92.1% over ARIMA, 48.8% over LR, 69.5% over SVR, 82.7% over KNN, and 57.3% over Ridge. In terms of RMSE, T2SNET outperforms ARIMA by 91.2%, LR by 54.7%, SVR by 70.8%, KNN by 81.2%, and Ridge by 58.4%. For MAPE, T2SNET improves by 92.8% over ARIMA, 48.0% over LR, 67.3% over SVR, 84.3% over KNN, and 58.8% over Ridge. Compared to the optimal deep learning baseline model CEEMDAN-RF-LSTM, T2SNET exhibits varying degrees of improvement in prediction accuracy. For example, on the university dormitory dataset, T2SNET outperforms the CEEMDAN-RF-LSTM model by 2.92%, 5.05%, and 3.30% in MAE, RMSE, and MAPE, respectively. On the university classroom dataset, T2SNET shows improvements of 4.56%, 9.45%, and 3.16% in these metrics. These experimental comparisons validate the absolute advantage of the T2SNET prediction model in forecasting building energy consumption tasks and reflect the practical application value of T2SNET in industrial scenarios.

#### 5.5.2 Case visualization.

**Fig 6:**
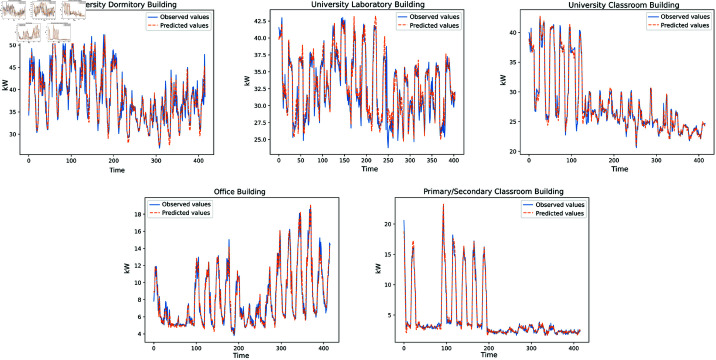
Performance of the T2SNET model on five different building energy consumption test datasets. The blue curve represents the observed values, while the orange curve represents the predicted values. The x-axis denotes the length of the test dataset, and the y-axis represents the energy consumption values.

To further demonstrate the performance of the T2SNET prediction model proposed in this study, Fig 6. presents the fitting between the predicted results of T2SNET and the actual observed values. From the visual examples, three interesting findings can be summarized:

1. The model’s predicted values fit the observed values accurately and consistently;
2. The model maintains satisfactory performance at the extremes of each day (maximum or minimum energy consumption);
3. The model’s performance does not decline due to fluctuations in energy consumption values.

These findings demonstrate that the T2SNET prediction model proposed in this study is highly robust, applies to forecasting energy consumption scenarios in various buildings, and overcomes the energy consumption data oscillation issue. Therefore, the proposed prediction model is more scientific and practical in real-world scenarios.

#### 5.5.3 Ablation experiment.

To comprehensively evaluate the impact of core components in our proposed framework, we conduct systematic ablation studies through four experimental configurations: (1) Full Model integrating CEEMDAN for multivariate sequence decomposition (humidity and energy), Twin Network for temporal feature embedding, and adaptive fusion gates; (2) Without CEEMDAN, which removes the decomposition module while retaining the Twin Network architecture to isolate sequential feature extraction capabilities; (3) Without Twin Network, preserving CEEMDAN preprocessing but eliminating temporal embedding layers to assess structural learning contributions; and (4) Baseline Configuration excluding both advanced components, relying solely on adaptive fusion gates and fully connected layers for fundamental prediction.

As quantitatively demonstrated in Table 6, the ablation study systematically validates the necessity of CEEMDAN decomposition and dual-TCN architecture in T2SNET. Disabling both components (Baseline) yields MAEs of 2.15 (dormitory) and 1.24 (laboratory), with MAPEs deteriorating to 8.92% and 12.37%, respectively. Isolating CEEMDAN decomposition (w/o Twin) reduces dormitory RMSE by 27.0% (2.78 to 2.03) by disentangling non-stationary load sequences into intrinsic mode functions (IMFs), where high-frequency IMFs capture transient equipment spikes and low-frequency IMFs model baseline consumption. The dual-TCN network alone (w/o CEEMDAN) improves laboratory MAE by 16.9% (1.24 to 1.03) through parallel causal convolutions: the primary TCN extracts long-term dependencies via dilated kernels, while the twin TCN refines local temporal patterns using dense convolutions. The full model achieves synergistic performance – dormitory/laboratory MAEs decrease to 1.33/0.54 (38.1%/56.5% reduction) – demonstrating that CEEMDAN’s noise-robust decomposition enhances the dual-TCN’s multiscale feature extraction, while the TCNs’ hierarchical gradients conversely optimize IMF reconstruction fidelity through backpropagation.

**Table 6:** Ablation study on the impact of CEEMDAN and temporal twin network.

Configuration	CEEMDAN Decomposition	Temporal Twin Network	University Dormitory			University Laboratory		
			MAE	RMSE	MAPE (%)	MAE	RMSE	MAPE (%)
Baseline	×	×	2.15	2.78	8.92	1.24	1.65	12.37
w/o CEEMDAN	×	✓	1.89	2.41	7.15	1.03	1.39	9.84
w/o Twin	✓	×	1.62	2.03	5.82	0.87	1.17	7.53
Full Model	✓	✓	**1.33**	**1.67**	**3.40**	**0.54**	**0.74**	**4.20**

## 6 Conclusion

The study develops T2SNET, a new model for building energy consumption prediction. The model separately processes energy consumption and meteorological data using a twin causal convolutional network, capturing important temporal relationships. It also integrates an embedding learning module to incorporate multi-scale time variables into feature extraction and prediction. Additionally, a multi-source data fusion module adaptively combines energy and meteorological features, improving prediction accuracy. The experimental results show that T2SNET outperforms traditional methods on multiple building energy consumption datasets. The model demonstrates strong generalization ability in practical applications, maintaining stable performance even in extreme cases. These findings highlight its potential for real-world energy management. Future research can explore the spatial relationships between buildings, analyzing energy consumption similarities and correlations across different structures. Incorporating spatial dependencies may further enhance prediction accuracy and support more efficient energy management strategies.

## Supporting information

S1 File T2SNET-Pro-master (ZIP)
