# Supplementary figures and images for: A novel twin time series network for building energy consumption predicting
Source: PLoS One. 2025 Jun 26;20(6):e0326576. doi: 10.1371/journal.pone.0326576 (PMC12200849; doi:10.1371/journal.pone.0326576)

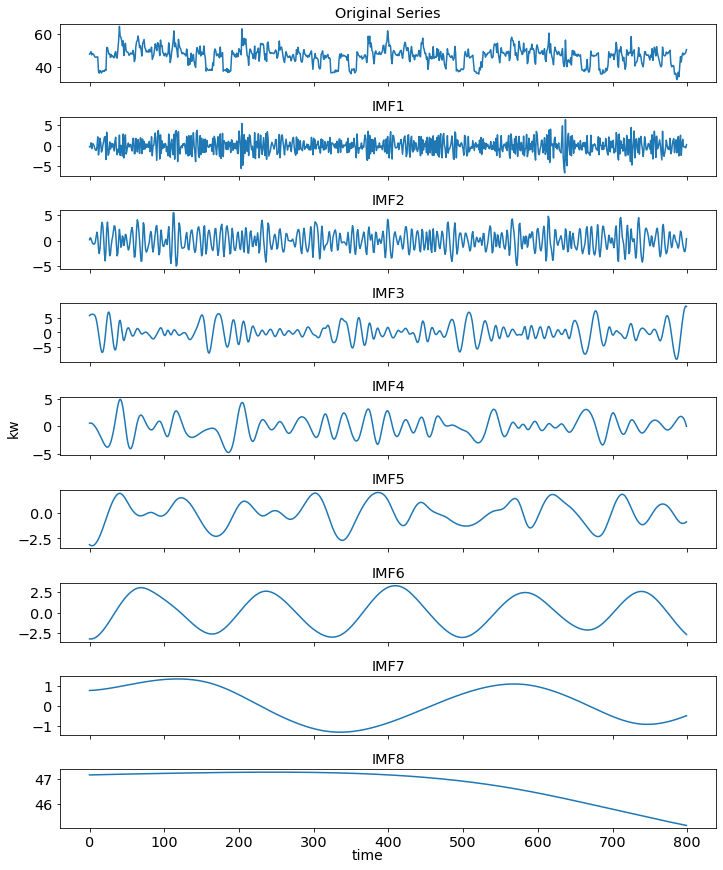

Supplement: S1 File — (ZIP) [file pone.0326576.s001.zip › T2SNET-Pro-master/T2SNET/baselines/Figures/CEEMDAN results for university laboratory.png]

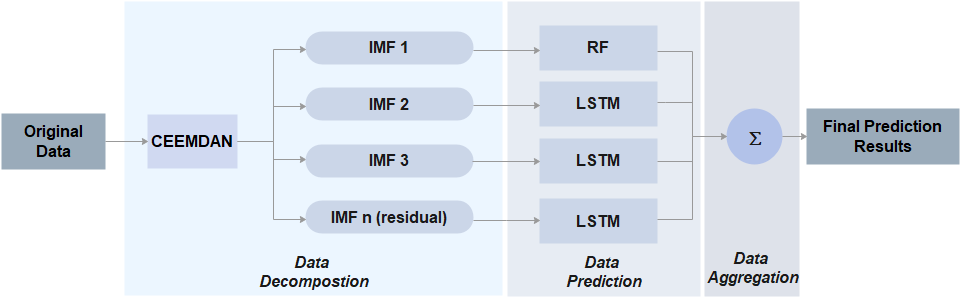

Supplement: S1 File — (ZIP) [file pone.0326576.s001.zip › T2SNET-Pro-master/T2SNET/baselines/Figures/Framework.png]

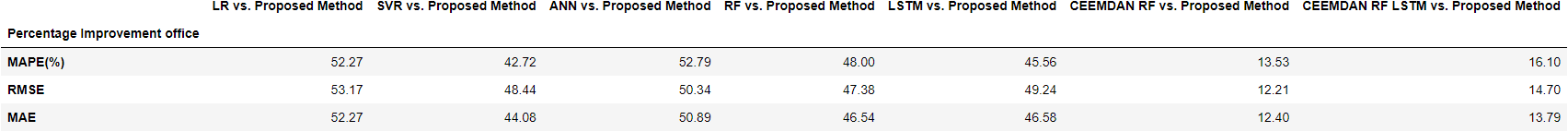

Supplement: S1 File — (ZIP) [file pone.0326576.s001.zip › T2SNET-Pro-master/T2SNET/baselines/Figures/PI_office_table.png]

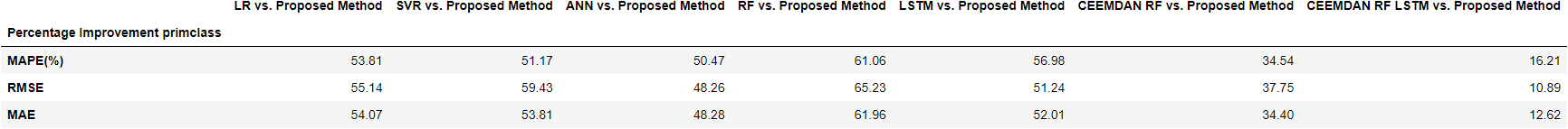

Supplement: S1 File — (ZIP) [file pone.0326576.s001.zip › T2SNET-Pro-master/T2SNET/baselines/Figures/PI_primclass_table.png]

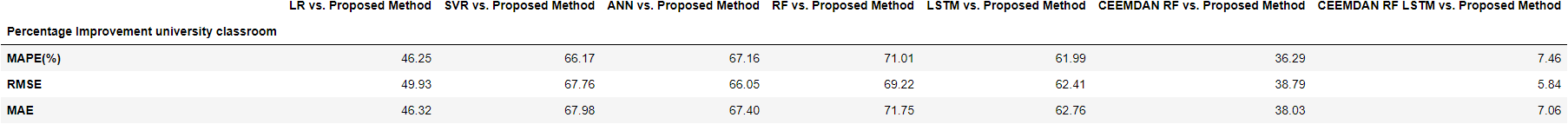

Supplement: S1 File — (ZIP) [file pone.0326576.s001.zip › T2SNET-Pro-master/T2SNET/baselines/Figures/PI_univclass_table.png]

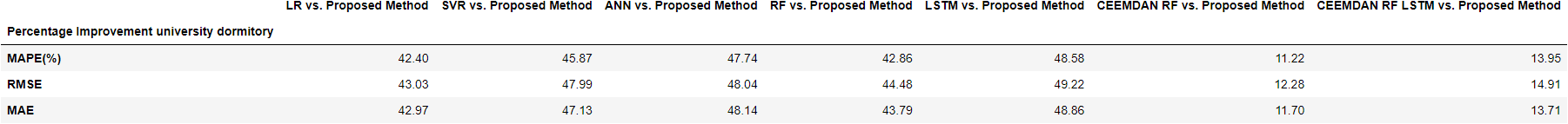

Supplement: S1 File — (ZIP) [file pone.0326576.s001.zip › T2SNET-Pro-master/T2SNET/baselines/Figures/PI_univdorm_table.png]

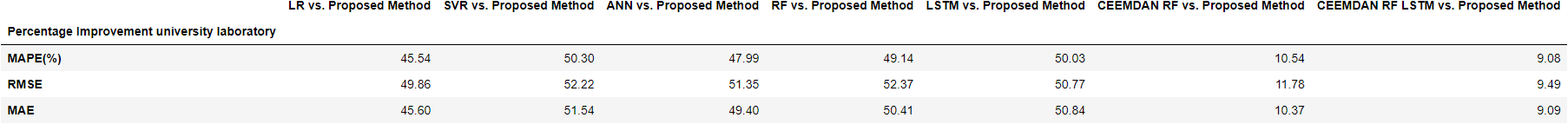

Supplement: S1 File — (ZIP) [file pone.0326576.s001.zip › T2SNET-Pro-master/T2SNET/baselines/Figures/PI_univlab_table.png]

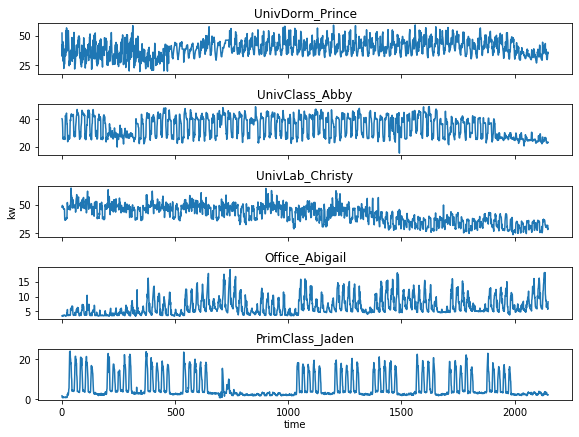

Supplement: S1 File — (ZIP) [file pone.0326576.s001.zip › T2SNET-Pro-master/T2SNET/baselines/Figures/hourly energy consumption from five buildings.png]

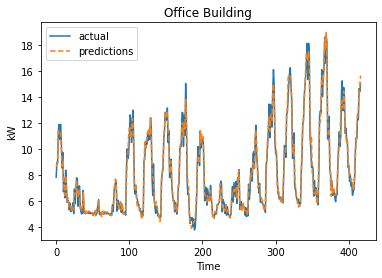

Supplement: S1 File — (ZIP) [file pone.0326576.s001.zip › T2SNET-Pro-master/T2SNET/baselines/Figures/plot office results.png]

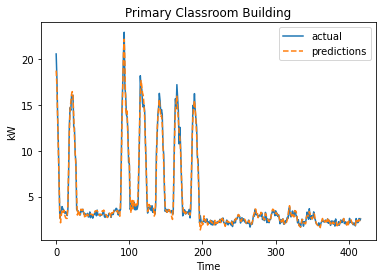

Supplement: S1 File — (ZIP) [file pone.0326576.s001.zip › T2SNET-Pro-master/T2SNET/baselines/Figures/plot primclass results.png]

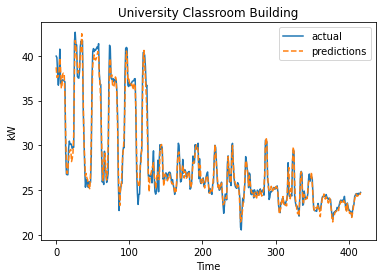

Supplement: S1 File — (ZIP) [file pone.0326576.s001.zip › T2SNET-Pro-master/T2SNET/baselines/Figures/plot univclass results.png]

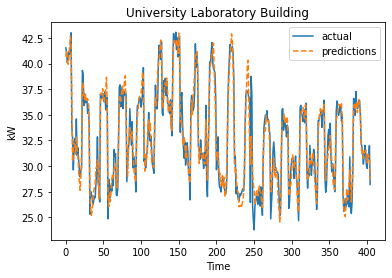

Supplement: S1 File — (ZIP) [file pone.0326576.s001.zip › T2SNET-Pro-master/T2SNET/baselines/Figures/plot univlab results.png]

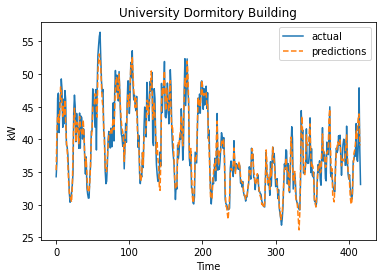

Supplement: S1 File — (ZIP) [file pone.0326576.s001.zip › T2SNET-Pro-master/T2SNET/baselines/Figures/plot_univdorm_results.png]
